# Oxidative Stress and Antioxidant Defense in Endometriosis and Its Malignant Transformation

**DOI:** 10.1155/2015/848595

**Published:** 2015-06-21

**Authors:** Takuya Iwabuchi, Chiharu Yoshimoto, Hiroshi Shigetomi, Hiroshi Kobayashi

**Affiliations:** ^1^Department of Research and Development, Metallogenics Co., Ltd., Chiba 260-0856, Japan; ^2^Department of Obstetrics and Gynecology, Nara Medical University, Nara 634-8522, Japan

## Abstract

The aim of this study was to investigate the role of redox status in endometriosis and its malignant transformation. A search was conducted between 1990 and 2014 through the English language literature (online MEDLINE PubMed database) using the keywords endometriosis combined with malignant transformation, oxidative stress, and antioxidant defense. In benign endometriosis, autoxidation and Fenton reaction of hemoglobin from the ferrous Fe^2+^ (oxyhemoglobin) state to the ferric Fe^3+^ (methemoglobin) state lead to production of excess reactive oxygen species (ROS) such as O_2_^−^ and ^∙^OH. Hemoglobin, heme, and iron derivatives in endometriotic cysts cause distortion in the homeostatic redox balance. Excess oxidative stress could trigger DNA damage and cell death. In contrast, endometriosis-associated ovarian cancer (EAOC) might be associated with an effective antioxidant defense, including heme oxygenases, cytochrome P450 family, and glutathione transferase family. The pattern of redox balance supports that enhanced antioxidants may be involved in the pathogenesis of malignant transformation. In conclusion, oxidant/antioxidant balance function is a double-edged sword, promoting cell death or carcinogenesis. Upregulation of antioxidant functions in endometriotic cyst may result in restoration of cell survival and subsequent malignant transformation.

## 1. Introduction

Endometriosis is one of the most common gynecologic diseases in women of reproductive age [1]. This disorder causes diverse progressive symptoms such as dysmenorrhea, infertility, and rarely malignant transformation. The clinical symptoms might be associated with repeated episodes of hemorrhage. During menstruation, chronic inflammation and oxidative stress occur in the pelvis, peritoneum, and ovary. Female reproductive system is vulnerable to the harmful effects of reactive oxygen species (ROS) that damage proteins, lipids, and DNA structure. The net emission of ROS results from the balance between free radical production (prooxidative process) and its elimination by antioxidants (antioxidant defense process) [2]. Oxidative stress is a key factor for progression of endometriosis. Many theories have been elaborated so far to clarify endometriosis pathogenesis. It is a multifactorial disease resulting from the complex interplay of several factors, including epigenetic alterations, genetic mutations, chromosomal imbalances, and hormonal and environmental risk factors, such as chronic inflammation and oxidative stress [1].

In conditions of pathological hemorrhage and hemolysis in the endometriotic cysts, cell-free hemoglobin is massively released from erythrocytes into the cyst fluid space. Erythrocytes are vulnerable to the harmful effects of ROS through several processes of hemoglobin and heme degradation [3]. A marked rise in oxidative stress has been implicated in endometriotic cell apoptosis. Paradoxically, it has been hypothesized that hemoglobin, heme, and iron-rich environment might be associated with increased mutagenesis, which can in turn drive the cell to cancer [4, 5]. An altered balance between prooxidant and antioxidant activities may have an impact on malignant transformation of endometriosis. The clinicopathological studies have suggested a malignant transformation of ovarian endometriosis to endometrioid and clear cell carcinomas [5]. The malignant processes may also include other Müllerian-type tumors, including Müllerian-type mucinous borderline tumor and serous borderline tumor and sarcomas such as adenosarcoma and endometrial stromal sarcoma in the female pelvic cavity [5].

Here, we attempt to integrate the recent advances in our understanding of the control of the hemoglobin, heme, and iron-induced redox balance in endometriosis and its malignant transformation, for example, endometriosis-associated ovarian cancer (EAOC).

## 2. Search Strategy and Selection Criteria

A review of the literature was conducted in order to investigate the redox balance in endometriosis and its malignant transformation. A MEDLINE search was performed using the key words “endometriosis”, “ovarian cancer”, “clear cell carcinoma”, “oxidative stress”, “antioxidants”, “ferrous”, and “ferric”. English language publications in PubMed and references from relevant articles published between 1990 and 2014 were analyzed. References in the studies identified were also searched. There were 780 articles identified by search. About 130 articles were potentially relevant. Thirty-seven publications available for the pathogenesis of endometriosis-associated malignant transformation were chosen based on the final selection taking into account the title and the summary analysis. Others were excluded due to various reasons, including selection bias, detection bias, reporting bias, and other possible sources of bias.

## 3. Hemoglobin, Heme, and Iron-Induced Oxidative Stress

After bleeding and hemolysis in endometriosis, the prooxidant hemoglobin transfers heme and iron derivatives to endometriotic lesions. Total iron is composed of heme iron and nonheme iron (free iron). Plasma iron binds to transferrin and is required for the de novo synthesis of erythrocytes. Iron plays a critical role in the reversible and stable binding of oxygen to hemoglobin [6]. Approximately two-thirds of the total body iron content is bound to hemoglobin contained in erythrocytes. Thus, body iron has significant beneficial effects on tissue homeostasis.

**Figure 1:**
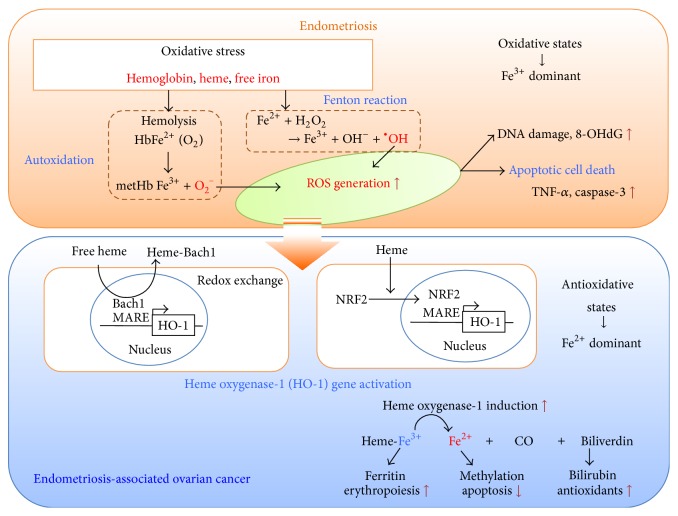
Malignant transformation of endometriosis: a fine-tuned balance between the formation of oxidants and the availability of endogenous antioxidants. Cell-free hemoglobin, heme, and iron massively released into the endometriotic cyst fluid space during menstruation are prone to autoxidation and may spontaneously convert oxyHb to metHb. ROS (O_2_^−^) are continuously generated by the autoxidation of hemoglobin. Iron derivatives also stimulate Fenton reaction, contributing to the generation of ROS (^∙^OH) in endometriotic cyst. Furthermore, hemoglobin and heme activate expression of a variety of antioxidant genes. Heme stimulates antioxidant HO-1 gene expression through direct binding to Bach1 or induction of NRF2 gene. Antioxidant is considered to be a double-edged sword. Excess ROS cause cell death. Antioxidants alleviate cell death by scavenging ROS (O_2_^−^ and ^∙^OH), allowing for increased cell survival and then carcinogenesis.

Once released from hemoglobin, however, free heme and iron are considered as generally toxic compounds. They oxidize most of the biomolecules including DNA, proteins, lipids, or cells through naturally occurring processes, autoxidation, and Fenton reaction. Both reactions contribute to the generation of ROS in endometriotic cyst (Figure 1).

### 3.1. Autoxidation

The nonenzymatic process of hemoglobin degradation is initiated by hydrogen peroxide or other ROS. Hemoglobin oxidization occurs slowly, which is referred to as “autoxidation” [3]. Hemoglobin is oxidized from the ferrous (Fe^2+^) oxygenated form (oxyHb-Fe^2+^) to the ferric (Fe^3+^) metform (metHb-Fe^3+^) with generation of the superoxide anion (O_2_^−^) as an autoxidation as follows:

(1) Hb-Fe2+oxyHb+O2⟶Hb-Fe2+-O2⟶Hb-Fe3+metHb+O2−

Hemoglobin autoxidation also produces harmful ROS such as hydrogen peroxide (H_2_O_2_) as by-products [7]. The oxidation reaction causes a transition from the deoxyHb to the metHb. Hydrogen peroxide reacts with the deoxyHb or metHb and consequently forms the highly reactive ferryl hemoglobin intermediate [7].

### 3.2. Fenton Reaction

Moreover, the labile ferrous iron (Fe^2+^) catalyzes both the Fenton reaction and the lipid peroxidation, resulting in the generation of ROS [8]. Nontransferrin bound iron has obvious chance to generate the free reactive radicals and has been suggested as cause and consequence in a number of pathological conditions [9]. Generation of free oxygen radicals such as hydroxyl radical (^∙^OH) by iron has been well documented in Fenton chemistry and Haber-Weiss reaction as follows [7]:

(2) Fe2++H2O2⟶Fe3++OH−+OH∙

Superoxide radial is associated with the formation of Fe^2+^ and H_2_O_2_ and in turn produces ^∙^OH.

Since erythrocytes in the endometriotic cyst space tend to hemolyse, hemoglobin released is prone to autoxidation and may spontaneously convert oxyhemoglobin (oxyHb, ferrous Fe^2+^ form) to methemoglobin (metHb, ferric Fe^3+^ form) [10]. Iron exhibits the most common redox couple: ferrous (Fe^2+^) and ferric (Fe^3+^) irons. OxyHb and metHb contain the ferrous state iron and ferric state iron, respectively. Ferrous iron is chemically reactive species [11]. The altered expression of two hemoglobin derivatives, conversions of oxyHb to metHb, suggests a shift favorable to excessive generation of potentially damaging ROS, resulting in oxidant/antioxidant imbalance in women with endometriosis. Recent study showed that the total iron levels for endometriotic cyst fluids and sera were approximately 250 mg/L and 1 mg/L, respectively, showing that iron was significantly higher in endometriotic cyst fluids compared to sera [12].

## 4. Altered Expression of Oxidant-Antioxidant Status in Endometriosis

We evaluate the status of oxidative stress and antioxidant status in endometriosis. As shown in Figure 1, upper part, hemoglobin, heme, and iron derivatives lead to excessive production of ROS and turn harmful to cells [11]. ROS play a crucial role in mediating tumor necrosis factor- (TNF-) alpha-induced apoptosis possibly through mitogen-activated protein (MAP) kinase-dependent manner [13]. Caspase-3 and bcl-2 have also been involved in ROS-related apoptosis [14, 15]. These parameters may ensure the physiological environment required for the induction of apoptosis in endometriosis.

As shown in Figure 1, lower part, various antioxidants can decrease oxidative stress that was induced by ROS in malignant transformation of endometriosis. Potent endogenous antioxidant protection systems modify highly ROS into less toxic intermediates. Erythrocytes provide abundant antioxidant enzymes that catalyze the breakdown of H_2_O_2_ and O_2_^−^. Furthermore, iron-dependent prooxidant activity must be controlled via mechanisms regulated by ferritin, transferrin, transferrin receptors, cubilin (for internalization of the urinary Fe^3+^-transferrin complex), divalent metal transporter, iron transporters, and iron regulatory proteins [16]. In addition, the genes encoding detoxifying/antioxidant enzymes include the heme oxygenase- (HO-) 1, the superoxide dismutases (SOD), the glutathione peroxidases (GPX), peroxiredoxins (PRX), catalase (CAT), and nuclear factor, erythroid 2-like 2 (NFE2L2) [17]. These cytoprotective genes function as the first protective barriers against oxidative stress.

In general, oxidants and antioxidants remain in balance. This balance functions as a double-edged sword deciding the cell fate, death, or carcinogenesis. Understanding the role of iron homeostasis and redox status in endometriotic cyst fluid may provide the molecular mechanism through which ROS modulate cellular processes such as cell death (degeneration and apoptosis; Figure 1, upper part) or carcinogenesis (proliferation and survival; Figure 1, lower part). The well-established link between the prooxidant/antioxidant imbalance and cancer will open new avenues for the study of the mechanisms that lead to carcinogenesis.

### 4.1. Heme Oxygenase- (HO-) 1 Induction by Heme and Oxidative Stress

Hemoglobin and heme activate expression of antioxidant defense-related genes. In particular, heme has been shown to bind to several transcription factors, including Bach1 (BTB and CNC homology 1, basic leucine zipper transcription factor 1) and Bach2. When Bach1 forms a heterodimer with MafK (v-maf avian musculoaponeurotic fibrosarcoma oncogene homolog K), the Bach1-MafK complex functions as a repressor of Maf recognition element (MARE) and HO-1 gene [18]. Therefore, heme inhibits the Bach1-induced repression of HO-1 gene expression by directly binding to Bach1 (Figure 1, lower part). From the clinical point of view, heme induces HO-1 expression in a number of diseases including endometriosis [19], intracerebral hemorrhage [20], or atherosclerotic intraplaque hemorrhage [21].

In addition, HO-1 is strongly induced by a variety of stimuli including oxidative stress, ROS, free heme, heavy metals, cytokines, some hormones, and ultraviolet light through the major transcription factor, NRF2, also known as NFE2L2 (nuclear factor, erythroid 2-like 2) [22] (Figure 1, lower part). The NRF2-mediated protection is accompanied by induction of a wide variety of detoxification and antioxidant enzymes (NAD(P)H quinone oxidoreductase (NQO1), glutathione S-transferase (GST), glutamate cysteine ligase (GCL), peroxiredoxin I (PRX I), and GPX), production of antiapoptosis genes (BCL2-associated X protein [BAX], BCL2-associated agonist of cell death [BAD], and caspase-3 [CASP3]), attenuation of oxidative stress (HO-1 and early growth response-1 [Egr1]), and suppression of inflammatory responses (interleukin- (IL-) 1 beta, IL-6, and TNF-alpha) [23]. Cytokines that are upregulated after ischemia, like IL-10, can induce HO-1 gene expression [24]. Thus, NRF2 can protect cells and tissues from many environmental toxicants, carcinogens, ischemia, drugs, and inflammatory insults by increasing the expression of a number of cytoprotective genes.

Finally, free iron is released from heme through HO-1 activity after hemoglobin breakdown. This enzyme degrades the heme moiety and liberates biologically active products: inorganic ferrous iron, carbon monoxide (CO), and biliverdin which play a unique protective and antioxidant role as well as having anti-inflammatory and antiapoptotic properties [25]. The HO-1-induced cytoprotective effect requires the coexpression of ferritin. CO has been found to possess significant cytoprotective, anti-inflammatory, and antiapoptotic properties via guanylate cyclase activation, generation of cyclic guanosine 3′,5′-monophosphate (cGMP), and activation of cGMP-dependent protein kinases [26]. Both biliverdin and bilirubin are potent antioxidants via chemical scavenging of peroxy radicals and inhibition of lipid peroxidation.

### 4.2. Antioxidant Gene Expression in Endometriosis and Endometriosis-Associated Ovarian Cancer (EAOC)

We provide evidence that an imbalance between the formation of oxidants and the availability of endogenous antioxidants is a major determinant of endometriosis and its malignant transformation. In response to the heme and iron-induced oxidative stress, NRF2 gene regulates the expression of a wide variety of detoxification and antioxidant enzymes [23]. Several studies demonstrated, however, that expression of antioxidant genes, NQO1, GSTP1, and GPX, was decreased in endometriosis, suggesting downregulation of oxidative metabolism genes [27, 28]. Inactivating gene mutations, polymorphisms, and allelic variants of the detoxification genes, including cytochrome P450 family (CYP1A1 and CYP2D6) and GST family (GSTT1, GSTP1, and GSTM1), which are associated with impaired functions, may be implicated in endometriosis susceptibility [27, 28]. Immunohistochemical study showed that endometriosis had stronger nuclear 8-hydroxy-2′-deoxyguanosine (8-OHdG, an oxidative stress marker) expression than EAOC [29]. These data support the idea that the disruption of the NRF2-mediated antioxidant defense pathway is a critical step in the pathophysiology of endometriosis development.

An increased risk of cancer may be associated with oxidative DNA damage and genotoxicity [30]. It is possible that antioxidants alleviate cell death by scavenging ROS and free radicals, thus allowing for increased carcinogenesis. For example, GST is upregulated in ovarian cancer [31]. GPX is overexpressed in clear cell ovarian cancer, arising from endometriosis [32]. Upregulation of antioxidant enzyme NQO1 has been observed in a variety of cancers, including liver, thyroid, breast, colon, and pancreas [33]. NQO1 C609T gene polymorphisms influence the risk for the gastric cancer [34]. Peroxiredoxin (PRX) promotes cancer cell proliferation and invasion [35]. In general, antioxidant enzymes have been overexpressed in a variety of cancers, demonstrating that the oxidant/antioxidant balance leads collectively to low oxidative stress levels in cancer. Recent research has highlighted that overexpression of a series of antioxidant genes via NRF2 and low ROS levels have been associated with cancer formation [36]. Although increased ROS may actually be important for carcinogenesis, antioxidant genes overexpressed in tissue offer a proliferation-permissive environment and give cells an advantage for survival and growth.

## 5. Oxidants and Antioxidants: Two Sides of the Same Coin in the Physicochemical Properties of Malignant Transformation

Recent data provide new valuable information that oxidative stress is considered to be a causative link between carcinogenesis and the redox imbalance, ineffective repair of DNA damage, and acquisition of oncogenic mutations [37]. Inflammation, resulting from oxidative stress, promotes both cellular senescence and carcinogenesis. It is assumed that the heme- and iron-mediated oxidative stress occurs from repeated hemorrhage in endometriosis; then, these compounds oxidatively modify genomic DNA and induce DNA damage, which is likely to be involved in the endometriotic cell death, rather than malignant transformation [4]. The DNA repair machinery in endometriosis is largely responsible for the repair of oxidative stress-induced DNA damage [38]. Restoration of the oxidant/antioxidant imbalance leads to an equilibrium that actually reestablishes redox homeostasis. In a chronic oxidative environment, sublethal concentrations of free radicals could cause genomic instability and mutations, which are responsible for adaptation of cells to oxidative stress and their survival. Effective antioxidative function can in turn pave the way for the pathway of events that might lead to malignant transformation. Overproduction of ROS causes cell death in endometriosis, while potential antioxidants may be able to slow down the ROS-induced cell death, leading to endometriosis-associated ovarian carcinogenesis. Recent knowledge related to the oxidant/antioxidant balance has been integrated to expand the paradigm for understanding carcinogenesis following repeated hemorrhage at the endometriotic lesions.

## 6. Conclusion

We focus on the role of hemoglobin, heme, and iron derivatives in endometriosis and EAOC, trying to outline the potential of oxidant/antioxidant balance function in the pathogenesis of malignant transformation of endometriosis. A functional link between increased levels of iron and areas of disease pathology has been recognized, including many malignancies and neurodegenerative disorders such as Alzheimer's and Parkinson's diseases [11]. We discuss the results to date and future possibilities.

Firstly, in endometriosis, hemoglobin, heme, and iron derivatives are generated from hemolysis of erythrocytes and abnormally accumulated in endometriotic cysts or in peritoneal cavity [39]. The endometriotic cells are especially prone to DNA damage due to direct exposure to these derivatives promoting ROS generation. When a mammalian cell detects genotoxic DNA damage, it initiates the DNA damage response that avoids entry into mitosis and instead accelerates DNA repair. In contrast, augmented oxidant property with an excess of ROS permits damaged cells to induce apoptosis and cell death. We consider that excess oxidative stress and possibly an insufficient repair mechanism may finally trigger the process of endometriotic cell death [40].

Secondly, the ability to survive the oxidative action of these derivatives appears to be more advantageous for endometriotic cell growth. The amount of ROS is counterbalanced by cellular antioxidant defense. Heme oxygenases, cytochrome P450 family, and GST family function as antioxidant enzymes. Some cells proliferate when the genome is susceptible to change or rearrangement. Other cells are able to adapt and survive in the environment with sublethal levels of oxidative stress, along with defective genomic repair, incomplete DNA replication, and genomic instability. Excess oxidative stress disrupts the normal cell function and has a role in cell death, while proper functioning of antioxidant defense may be crucial for the promotion of carcinogenesis. Antioxidants can adversely accelerate or contribute to malignant transformation of endometriosis.

Finally, each endometriotic lesion may display significant differences with regard to the level of responsiveness to free radicals or antioxidant defense. A significant excess of hemoglobin, heme, and iron derivatives initializes the oxidative processes and then leads to cell death. An enhanced antioxidation capability or sublethal levels of ROS exhibit cytoprotective properties and can protect endometriotic cells from cell death or apoptosis but lead to accumulation of DNA damage, aneuploidy, genomic instability, and mutations [41]. In this situation, an enhanced antioxidation capability may act as a trigger for cell survival and subsequent carcinogenesis. Interestingly and somewhat paradoxically, our review revealed that decreased oxidant status and increased antioxidant capacity may have a crucial role in the pathogenesis of EAOC, suggesting that hemoglobin, heme, and iron derivatives can be considered as molecular switches.

The perturbation of the oxidant/antioxidant balance or aberrant redox homeostasis could be a common mechanism for progression of a growing number of malignancies, including malignant mesotheliomas [42], hepatocellular carcinoma [43], renal cell carcinoma [44], and endometriosis-associated ovarian carcinoma [45], as well as major neurodegenerative diseases, of particular relevance in Alzheimer's disease [46], Parkinson's disease [47], and amyotrophic lateral sclerosis [47]. These disorders are considered to be the consequence of fine-tuned imbalance between oxidants and endogenous antioxidants [5].

In conclusion, our goal is to outline a plausible scenario in which antioxidant environment contributes to subsequent malignant transformation in women with endometriosis. There is evidence that hemoglobin, heme, and iron derivatives may represent a part of the missing link between cell death and carcinogenesis and can be considered as possible EAOC pathogenesis instigators. The oxidant/antioxidant imbalance might present a dual role, cell death and carcinogenesis, as a double-edged sword.
